# Pan-Genome Analysis of *TIFY* Gene Family and Functional Analysis of *CsTIFY* Genes in Cucumber

**DOI:** 10.3390/ijms25010185

**Published:** 2023-12-22

**Authors:** Kun Liu, Haiyu Xu, Xinbin Gao, Yinghao Lu, Lina Wang, Zhonghai Ren, Chunhua Chen

**Affiliations:** 1 College of Horticulture Science and Engineering, Shandong Agricultural University, Tai’an 271018, China; 2021110296@sdau.edu.cn (K.L.); 2021110306@sdau.edu.cn (H.X.); 2022120415@sdau.edu.cn (Y.L.); lnwang@sdau.edu.cn (L.W.); zhren@sdau.edu.cn (Z.R.); 2 College of Horticulture, Northwest A and F University, Yangling 712100, China; xinbingao2023@163.com

**Keywords:** cucumber, TIFY, gray mold, disease resistance

## Abstract

Cucumbers are frequently affected by gray mold pathogen *Botrytis cinerea*, a pathogen that causes inhibited growth and reduced yield. Jasmonic acid (JA) plays a primary role in plant responses to biotic stresses, and the jasmonate-ZIM-Domain (JAZ) proteins are key regulators of the JA signaling pathway. In this study, we used the pan-genome of twelve cucumber varieties to identify cucumber *TIFY* genes. Our findings revealed that two *CsTIFY* genes were present in all twelve cucumber varieties and showed no differences in protein sequence, gene structure, and motif composition. This suggests their evolutionary conservation across different cucumber varieties and implies that they may play a crucial role in cucumber growth. On the other hand, the other fourteen *CsTIFY* genes exhibited variations in protein sequence and gene structure or conserved motifs, which could be the result of divergent evolution, as these genes adapt to different cultivation and environmental conditions. Analysis of the expression profiles of the *CsTIFY* genes showed differential regulation by *B. cinerea*. Transient transfection plants overexpressing *CsJAZ2*, *CsJAZ6*, or *CsZML2* were found to be more susceptible to *B. cinerea* infection compared to control plants. Furthermore, these plants infected by the pathogen showed lower levels of the enzymatic activities of POD, SOD and CAT. Importantly, after *B. cinerea* infection, the content of JA was upregulated in the plants, and cucumber cotyledons pretreated with exogenous MeJA displayed increased resistance to *B. cinerea* infection compared to those pretreated with water. Therefore, this study explored key *TIFY* genes in the regulation of cucumber growth and adaptability to different cultivation environments based on bioinformatics analysis and demonstrated that *CsJAZs* negatively regulate cucumber disease resistance to gray mold via multiple signaling pathways.

## 1. Introduction

Throughout their lifecycle, plants often encounter various abiotic and biotic stresses, such as drought, salt, temperature stress and infection by pathogens (bacterial, fungal, or oomycete and so on) [1,2,3,4]. Plant transcription factors (TFs), including members of the NAC, MYB, WRKY, TIFY, AP2/ERF, and bZIP families, act as important components in plant tolerance against various stresses by mediating plant physiological and biochemical processes [5,6,7,8,9,10,11].

The TIFY gene family consists of plant-specific TFs that contain a highly conserved motif TIF[F/Y]XG. This motif is located within a TIFY domain, which spans approximately 36 amino acids (aa). According to the domain structure, they can be divided into four subfamilies, namely, TIFY, PEAPOD (PPD), jasmonate-ZIM-domain (JAZ), and ZIM-like (ZML) [12,13]. Among them, PPD, JAZ, and ZML subfamilies contain more than one domain. Except for the TIFY domain, the PPD subfamily proteins contain a PPD domain and a truncated JA-associated (Jas, also named CCT-2) domain lacking the conserved Proline-Tyrosine (PY) at the C-terminus [14]; the JAZ subfamily contains a Jas domain [15]; the ZIM/ZML subfamily contains a CCT (CONSTANS, CO-like and TOC1) domain and a C2C2-GATA zinc-finger domain [16,17]. Until now, the identification of *TIFY* genes has been performed in many species, including *Arabidopsis thaliana* [17], *Glycine soja* [18], *Solanum lycopersicum* [19], *Brassica oleracea*, *Pyrus pyrifoli* [20], *Oryza sativa* [21], *Triticum aestivum* [22], and *Zea mays* [12].

A number of studies have demonstrated that TIFY TFs are involved in plant developmental processes and hormonal responses. For example, *TdTIFY11a* was highly induced by salt treatment, and over-expressing *TdTIFY11a* promoted the germination and growth rates of wheat plants under high-salinity conditions [22]. The JAZ genes are perhaps the best-characterized members, and they seem to play a crucial role in the pathway of jasmonic acid (JA) [15,23]. JA is well known as the hormone that regulates plant defenses to biotic stresses (such as necrotrophic pathogens, fungi, insect and nematodes) and abiotic stresses (such as wounding, UV light, and water deficit) [24,25,26]. In *Arabidopsis*, the TFs MYC3 and MYC4 are two targets of JAZ repressors, and they contribute to the activation of JA-dependent defenses against *Spodoptera littoralis.* Contrary to MYC3 and MYC4, which trigger a strong defense response to *S. littoralis*, single *myc3* and *myc4* mutants showed an enhanced resistance to the hemibiotrophic pathogen *Pseudomonas syringae pv tomato* DC3000 [9]. The *JAZ7* activation-tagged *Arabidopsis* mutant showed increased susceptibility to the fungal pathogen *Fusarium oxysporum* [27]. Additionally, JAZ7-overexpressing plants exhibited a strong drought-tolerance phenotype [28]. In addition, JAZs could directly interact with the MYB family in *Rosa chinensis*. JAZ1, a key repressor of JA signaling, directly interacts with RcMYB84, and this JAZ1-RcMYB84 complex binds to the promoter of RcMYB123, inhibiting its transcription. When treatment with JA, JAZ1 was degraded, RcMYB84 and RcMYB123, which activate the plant’s defense responses against fungal pathogen *Botrytis cinerea*, were released [29]. In soybean, overexpression of a TIFY family gene, *GsJAZ2*, enhanced tolerance to alkaline stress [30].

Cucumber (*Cucumis sativus* L.), an economically important vegetable crop, is an annual climbing plant and produces edible tender fruits. Cucumbers are commonly cultivated in greenhouses due to their preference for warm temperatures. Because of the high humidity in greenhouses, they frequently encounter many different types of pathogens, including bacterial, viral, fungal, and oomycete, that severely prevent growth and may have a great impact on production [31,32,33]. Of these, *Botrytis cinerea* is the causal agent of gray mold, which is one of the top 10 fungal plant pathogens and causes severe damage, both pre- and post-harvest. *B. cinerea* is a necrotrophic pathogen with a broad host range, infecting more than 200 types of plants [34,35,36]. Previous studies suggested that enhancing the tolerance of cucumber cultivars was an efficient strategy for disease control [33]. Thus, studying the genes involved in the regulation of the gray mold pathogen resistance response is important for enhancing the economic value of cucumber production. Given that the TIFY TFs play an important role in regulating plant defenses and stress responses, there is growing interest in identifying functional *CsTIFY* genes that regulate resistance responses of cucumber plants against the infection of *B. cinerea.* Based on the cucumber 9930 genome v2.0, seventeen *CsTIFY* genes have been identified [37], but a comprehensive understanding of the TIFY family in different cucumber varieties remains incomplete.

There has been a growing awareness that single reference genomes do not reflect the diversity within a species [38,39,40,41]. Therefore, the pan-genome, originally proposed in bacteria, is now widely used in plant, fungal, and animal genomics to assess genetic diversity within species [39,40,41,42]. In cucumber, a graph-based pan-genome was built by analyzing twelve chromosome-scale genome assemblies [43]. In this study, a new genome-wide identification of *TIFY* genes was performed using this cucumber pan-genome. Furthermore, we investigated the crucial genes involved in the response to gray mold in cucumber, offering potential for developing resistant cucumber varieties against gray mold disease.

## 2. Results

### 2.1. Identification of TIFY Genes Based on Cucumber Pan-Genome

**Figure 1:**
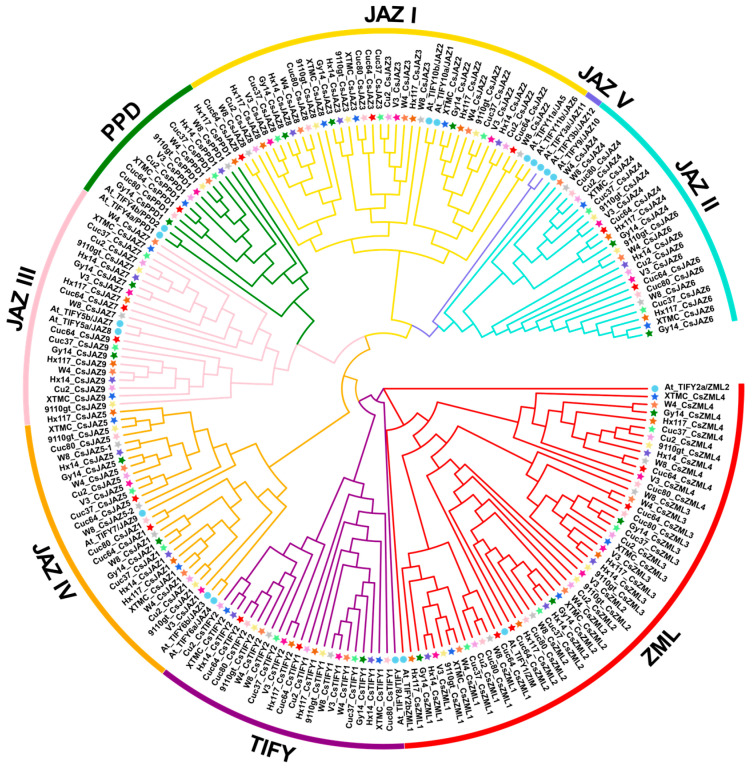
The phylogenetic tree of total TIFY proteins from twelve cucumber varieties and *Arabidopsis* genomes. These proteins were phylogenetically analyzed using MEGA7 software (7.0.21) with 1000 bootstrap tests. The different colored arcs represent the eight subgroups of TIFY proteins. The different colored stars and blue circles represent TIFY proteins from twelve cucumber and *Arabidopsis*, respectively.

In a previous study, seventeen *TIFY* genes were identified in the cucumber 9930 genome v2.0 [37]. Given that a cucumber pan-genome was built by analyzing twelve cucumber varieties’ genome assemblies [43], the new identification of *CsTIFY* genes was performed based on this pan-genome. Consistent with the previous study, seventeen putative *TIFY* genes were identified in the cucumber 9930 genome v2.0 using a Hidden Markov Model (HMM) search with the TIFY domain (PF06200) (Table 1). These genes were confirmed to contain TIFY domains according to Pfam and SMART analysis. However, one *TIFY* gene named *CsJAZ6* in the previous study was removed due to the lack of a conserved TIFY domain. Additionally, *TIFY* gene *1G435720*, previously named *CsJAZ2*, was not identified in 9930 cucumber genome v3.0 and genomes of other cucumber varieties. The genome assembly of 9930 in v3.0 was of a higher quality and more complete than that in v2.0. Therefore, fifteen *TIFY* genes identified in the genome v3.0 of cucumber 9930 were used in this study. There were sixteen *CsTIFY* genes obtained in XTMC, Cu2, Cuc37, Cuc64, W4, Hx117, and 9110gt (Table 1), which had one more gene, *CsJAZ9*, than in cucumber 9930. Additionally, fifteen *CsTIFY* genes were also identified in Hx14 and Gy14, including *CsJAZ9*, but lack CsJAZ4 and CsTIFY2, respectively. In cucumber W8, *CsJAZ9* was not found, but two *CsJAZ5* genes (5G044750 and UNG162140.1) were identified, named *CsJAZ5-1* and *CsJAZ5-2*, respectively. The number of *TIFY* genes in cucumber Cuc80 was the lowest, with only thirteen *TIFY* genes identified (Table 1). To construct a phylogenetic tree, the amino acid sequences of TIFY TFs from *Arabidopsis* and twelve cucumber varieties were used. In *Arabidopsis*, TIFY proteins were classified into eight groups, including TIFY, PPD, AML, and JAZ I-V [12]. As shown in Figure 1, cucumber TIFY proteins could be categorized into seven clades based on the classifications of TIFYs in *Arabidopsis*, with the exception of clade JAZ V, which was not observed in the TIFY proteins of cucumber.

In order to investigate whether there was genetic diversity for *CsTIFY* genes within different cucumber varieties, the lengths of CsTIFY proteins were analyzed. As shown in Table 2, ten *CsTIFY* genes showed diversity in protein length across these twelve cucumber varieties. For example, the protein length of CsJAZ1 ranges from 340 aa (in cucumber 9930) to 356 aa (in cucumber Cuc80 and Cuc64). The length of the *CsJAZ7* gene-encoded protein is 130 aa in cucumber Cuc64, W4, and W8, whereas it was 132 aa in other cucumber varieties. Only six *CsTIFY* genes, including *CsJAZ3*, *CsJAZ5*, *CsJAZ6*, *CsJAZ8*, *CsZML1*, and *CsTIFY1*, exhibited the same protein length across the twelve cucumber varieties (Table 2). Considering the high diversity of CsTIFY protein length in different cucumber variety (Table 2), we investigated allelic variation patterns for sixteen characterized *TIFY* genes. We identified 71 variants localized within these *CsTIFY* genes, comprising 56 single nucleotide polymorphisms (SNPs) and 15 insertions and deletions (InDels) (Table S1). We found that the longer length of CsJAZ1 in cucumber Cuc80 and Cuc64 was a result of 51 bp fragment insertion, whereas the shorter length of the CsJAZ7 in Cuc64, W4, and W8 was due to 6 bp fragment deletion. The sequences of *CsJAZ5* and *CsJAZ6* are highly conserved, with no observed variations across different cucumber varieties (Table 2 and Table S1). The genes *CsJAZ3, CsJAZ8, CsZML1*, and *CsTIFY1* harbor several SNPs; thus, the protein sequences varied among various cucumber varieties (Table S1 and Figure S1). The results indicate that *CsJAZ5* and *CsJAZ6* genes exhibited evolutionary conservation throughout the different cucumber varieties and might play an important role in cucumber growth.

### 2.2. Gene Structure and Motif Composition of CsTIFYs

**Figure 2:**
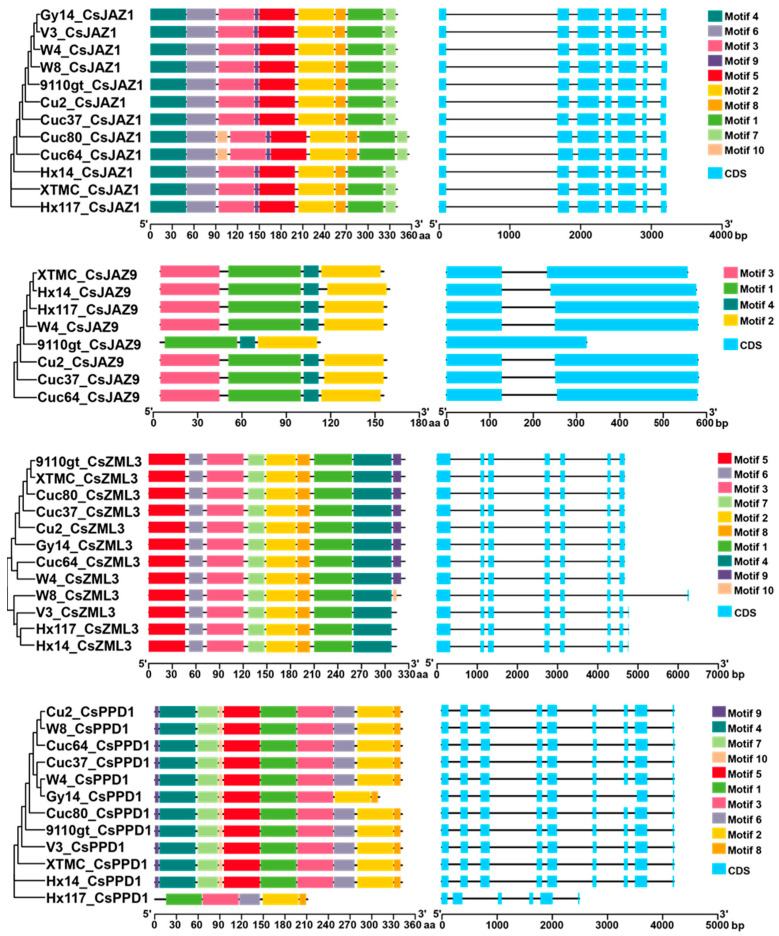
The phylogenetic tree, conserved protein motifs, and gene structure of *CsTIFY* genes, which vary in protein length in at least three different cucumber varieties. Right panel: gene structure, blue squares indicate CDS regions and black lines indicate introns. Middle panel: conserved protein motifs. The colorful boxes delineate different motifs. Left panel: the phylogenetic tree. The clustering is performed according to the results of phylogenetic analysis. CDS, coding sequence; aa, amino acid.

The diversity of gene structure can reflect the evolution of multigene families [44]. Therefore, TBtools software (v2.012) was used to analyze the exon–intron organization of the *CsTIFY* genes, which vary in the length of the amino acid sequence in at least three different cucumber varieties, including two *JAZ* genes, one *ZML* gene, and one *PPD* gene (Figure 2). Among them, the *CsJAZ9* gene, which encodes the protein length ranging from 107 (in cucumber 9110gt) to 154 aa (in cucumber Hx14 and Hx117), exhibited the lowest number of introns, with only one intron in all cucumber varieties, excluding cucumber 9110gt, which had none (Table 2 and Figure 2). We observed variations in the protein length of *CsJAZ1* (encoding a protein with a range of 339 to 356 aa) among different cucumber varieties. However, the gene structures remained consistent, containing seven exons in various cucumber lines (Table 2, Figure 2). We found that the *TIFY* genes *CsZML3* and *CsPPD1* have five to nine exons (Figure 2). Members of the same or similar protein length in different cucumber lines share the same intron/exon, while those with significant differences in protein length have distinct gene structures. For example, gene *CsPPD1* encodes a protein with a length ranging from 207 to 336 aa and contains eight introns in all cucumber varieties, except for cucumber Hx117 and Gy14, where it encodes a shorter protein (encoding 207 and 305 aa, respectively) with fewer introns (Figure 2). The results indicate that the gene structure of the same gene varies among different cucumber varieties, and this variation is correlated with the protein length, but is not identical.

To gain a deeper understanding of the conservation and diversification of these TIFYs, conserved motifs were identified using MEME motif analysis (Figures S1 and S2). As anticipated, *CsTIFYs* with varying protein lengths and gene structures displayed distinct motif compositions. *CsPPD1* in cucumber Gy14 and Hx117 exhibited shorter protein lengths, different gene structures, and decreased conserved motif number compared to those in other cucumber varieties (Table 2 and Table S2, Figure 2). Additionally, we noted that a *CsTIFY* gene with comparable protein lengths and identical gene structure displayed distinct motif compositions across different cucumber varieties. For example, the protein length of *CsJAZ1* gene was longer in the cucumber Cuc64 and Cuc80, with a protein length of 356 aa, compared to other varieties with protein lengths of 339 or 340 aa. Although the gene structure remained unchanged, the conserved motif increased, and Motif 10 was found to be specific in the cucumber Cuc64 and Cuc80 (Figure 2). Overall, this could be attributed to divergent evolution, as these genes in various cucumber lines undergo adaptations to distinct cultivation and environmental conditions.

Additionally, the exon–intron organization and conserved motifs of two *CsTIFY* genes (*CsJAZ5* and *CsJAZ6*), which code for proteins of the same sequence and length, were also studied. The results illustrated in Figure S2 indicate no differences in gene structure and conserved motifs. This indicates that these genes are evolutionarily conserved across different cucumber varieties and suggests their potential importance in cucumber growth.

### 2.3. Chromosome Distribution and Synteny Analysis of CsTIFY Gene Family

The genome sequence of cucumber 9930 has been the subject of numerous studies as the earliest variety to be sequenced. Hence, we chose *CsTIFY* genes from the cucumber variety 9930 as representatives for further study. As shown in Figure S3, fifteen TIFY family genes were not evenly dispersed across all seven chromosomes in cucumber 9930. Chromosome 2 harbored the highest number of *CsTIFY* genes (4), while only one was found on chromosome 4 and 5; additionally, two genes on chromosomes 1, 3, and 6, and three genes on chromosome 7.

Segment and/or tandem duplication always reflect the evolution of the plant genome and contribute to the expansion of the gene family [45]. In the analysis of *CsTIFY* genes, three pairs of duplicated genes were identified in the cucumber 9930 *TIFY* gene family: *CsTIFY1*/*CsTIFY2*, *CsJAZ2*/*CsJAZ8*, and *CsJAZ3*/*CsJAZ8* (Figure S3 and Table S3). Additionally, a tandem duplication event was observed in the cucumber *TIFY* genes, specifically in the *CsZML1*/*CsZML2* genes located within a chromosomal region of 200 kb (Figure S3 and Table S3). These findings suggest that some *CsTIFY* genes may have originated from both segmental and tandem duplications, indicating that segmental and tandem duplication events have played a role in the evolution of *CsTIFY* genes.

We further explored the phylogenetic mechanisms of the cucumber TIFY family by comparing it with other species, including three dicots (*Arabidopsis*, tomato, and melon) and two monocots (rice and maize) (Figure S4). A total of 9 gene pairs between cucumber and rice, 7 gene pairs between cucumber and maize, 16 gene pairs between cucumber and *Arabidopsis*/tomato, and 20 gene pairs between cucumber and melon were found, respectively (Figure S4 and Table S4). Cucumber and melon are both members of the gourd family. Our research revealed that over 85% of the *CsTIFY* genes show a syntenic relationship with *TIFYs* in melon, suggesting that the *TIFY* genes in cucumber and melon might evolve from the same ancient *TIFY* genes. Consistent with a previous study [37], the duplicate gene pairs all belong to the same subfamily, suggesting that distinct subfamilies were relatively conserved throughout evolution.

### 2.4. Responsive Analysis of CsTIFY Genes under Gray Mold Stress

**Figure 3:**
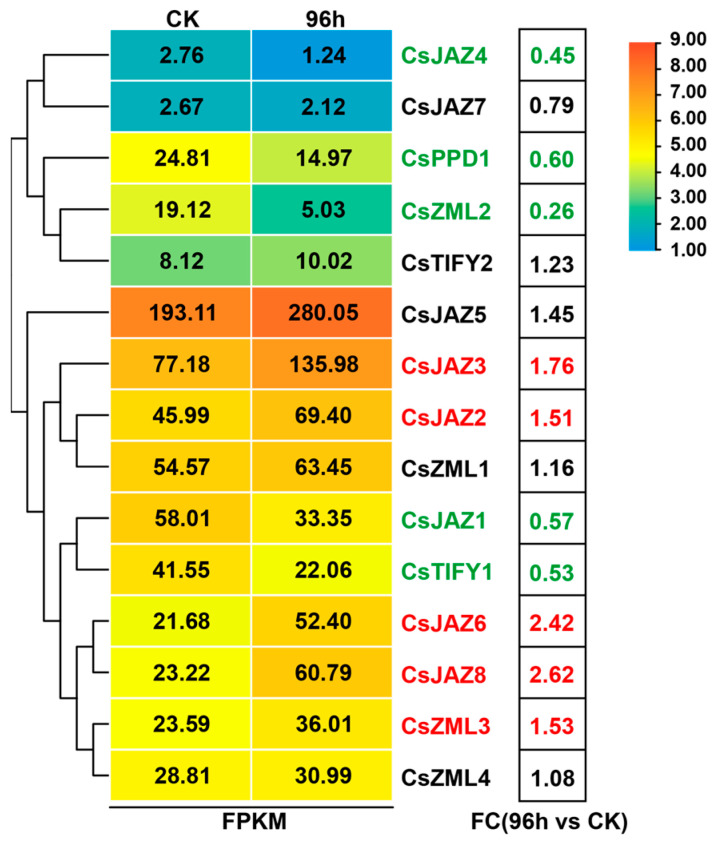
Heat map of *TIFY* gene expression under gray mold stress. The transcription of *TIFY* genes was determined at 96 hpi of *B. cinerea* in cucumber leaves, without inoculation as the control (CK). Gene names and fold-change in red indicate significantly upregulated genes, and those in green indicate significantly downregulated genes. FC, fold-change; h, hours.

Previous studies have shown that the expression of JAZ subfamily genes is significantly affected when cucumber plants are infected by *B. cinerea* [37]. In this study, we analyzed the expression pattern of *CsTIFY* genes using a public transcriptome of cucumber leaves inoculated with gray mold (*B. cinerea* strain B05.10). We found that out of the fifteen detected *CsTIFY* genes, ten of them showed differential expression compared to the control at 96 hours post-inoculation (hpi) of *B. cinerea* in cucumber leaves. Specifically, *CsJAZ2*, *CsJAZ3*, *CsJAZ6*, *CsJAZ8*, and *CsZML3* were upregulated, indicating their induction to play roles under gray mold stress. On the other hand, *CsJAZ1*, *CsJAZ4*, *CsTIFY1*, *CsPPD1*, and *CsZML2* exhibited decreased expression (Figure 3).

**Figure 4:**
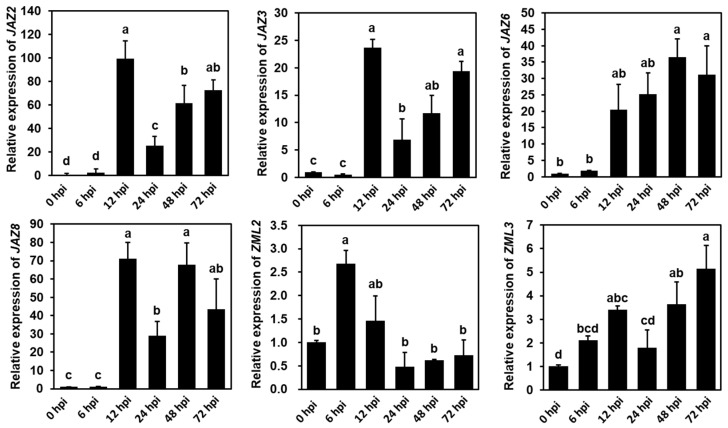
Expression pattern of *TIFY* genes after inoculation with *B. cinerea*. The error bars show the standard error of the mean of three biological replicates. Different lowercase letters indicate differences at *p* < 0.05.

To further investigate the expression of *CsTIFY* genes in plants following infection with the pathogen *B. cinerea*, we specifically selected six differentially expressed genes (DEGs) of *CsTIFYs* for analysis using qRT-PCR at 6, 12, 24, 48, and 72 hpi. These six genes consisted of five upregulated genes and one downregulated gene, namely *CsZML2*. The qRT-PCR results revealed that *CsZML2*, *CsJAZ2*, *CsJAZ3*, and *CsZML3* were initially upregulated and then downregulated, with the first peak of expression observed at 6 or 12 hpi. Additionally, *CsJAZ6* displayed a continuous upregulation from 6 to 72 hpi (Figure 4). These results suggest that these genes may play crucial roles in plant responses to pathogen-induced stress.

### 2.5. Functional Analysis of CsTIFY Genes in Resistance Response of Gray Mold

**Figure 5:**
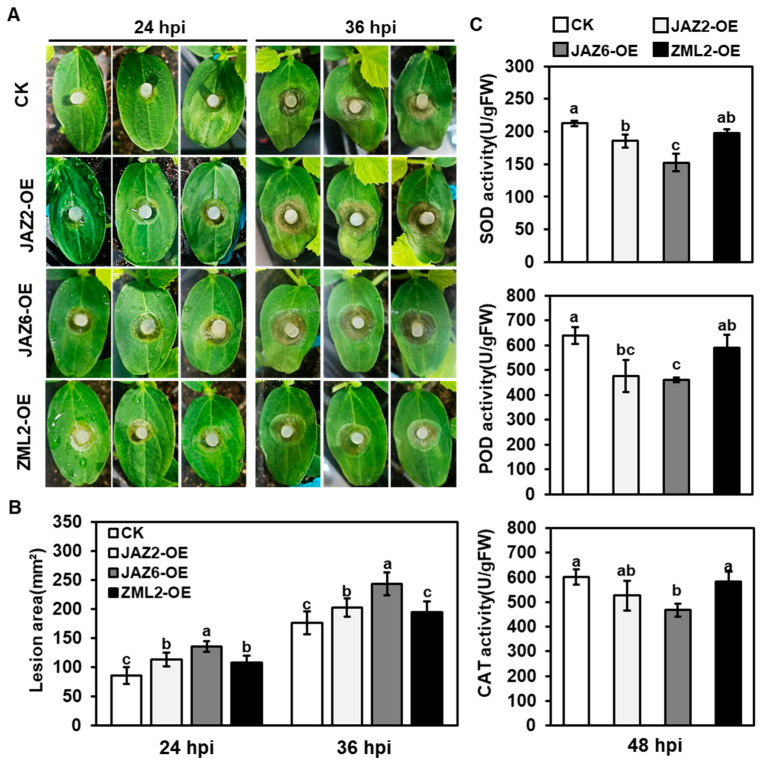
Analysis of disease symptoms and ROS accumulation in transient transfection plants inoculated with *B. cinerea*. (**A**) The symptoms of cucumber cotyledons inoculated with *B. cinerea*. (**B**) The lesion area of cucumber cotyledons inoculated with *B. cinerea*. (**C**) The enzymatic activities of SOD, POD and CAT after *B. cinerea* infection. Different lowercase letters indicate differences at *p* < 0.05; error bars indicate standard deviation; hpi, hours post inoculation.

Given *CsZML2*, *CsJAZ2*, and *CsJAZ6* with different expression patterns, we investigated the potential role of these three genes in responding to gray mold stress. Transient expression assays were used to transform *35S::CsZML2*, *35S::CsJAZ2*, *35S::CsJAZ6* and inoculation buffer (control) in cucumber cotyledons, respectively. Transient transfection cucumber seedlings were grown under normal conditions for approximately 18 hours (h) before being inoculated with *B. cinerea*. Among the seedlings, the cotyledons overexpressing *CsJAZ6* displayed the most severe disease symptoms, characterized by the largest necrotic plaques, compared to the cotyledons of the other seedlings (Figure 5A). Moreover, both *CsZML2* and *CsJAZ2* also reduced resistance to *B. cinerea* infection, resulting in larger necrotic plaques compared to the control seedlings (Figure 5A,B). Additionally, we examined the enzymatic activities in the reactive oxygen species (ROS) clearance system, such as SOD, POD, and CAT. After the inoculation treatment, transient transfection plants overexpressing *CsJAZ2* or *CsJAZ6* showed a significant decrease in POD, SOD, and CAT activities compared to the control (Figure 5C). These findings suggest that *CsZML2*, *CsJAZ2*, and *CsJAZ6* all have a detrimental impact on the cucumber defense response against the gray mold pathogen, with *CsJAZ6* potentially playing a particularly significant role. It can be inferred from these results that the overexpression of *CsJAZ2* and *CsJAZ6* could inhibit the defense resistance of cucumber by affecting the accumulation of ROS.

### 2.6. CsJAZs Regulate Resistance Response of Gray Mold via JA Pathway

**Figure 6:**
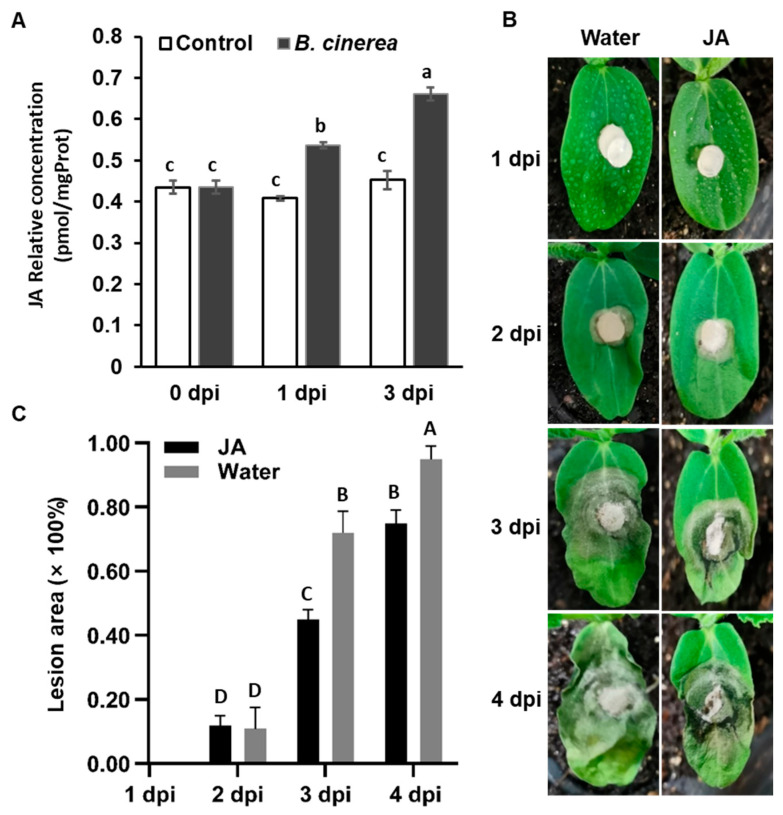
JA mediates cucumber resistance to *B. cinerea*. (**A**) Relative concentration of JA after inoculation with *B. cinerea*. (**B**) The symptoms of cucumber cotyledons pretreated with MeJA or water after *B. cinerea* inoculation. (**C**) The lesion area of cucumber cotyledons pretreated with MeJA or water after *B. cinerea* inoculation. Different letters indicate significant differences at *p* < 0.05; dpi, days post-inoculation.

JA is well known as the hormone that regulates plant defense responses to biotic stresses, with the *JAZ* genes playing a critical role in the JA signaling pathway. In order to explore the function of JA in regulating cucumber plants’ resistance to *B. cinerea*, we quantified JA levels in cucumber leaves after *B. cinerea* inoculation. Figure 6A illustrated a significant increase in JA content after *B. cinerea* inoculation compared to the control. Subsequently, cucumber cotyledons were pretreated with exogenous MeJA and water (control) in a consistent manner. Five hours after pretreatment, the cotyledons were inoculated with *B. cinerea*. The cotyledons of control seedlings displayed more severe disease symptoms, with larger lesion areas, compared to those pretreated with MeJA (Figure 6B,C). These results suggested that *CsJAZs* negatively regulate the JA pathway, thereby contributing to cucumber’s susceptibility to gray mold.

## 3. Discussion

### 3.1. Bioinformatics Analysis of CsTIFYs Based on Cucumber Pan-Genome

Although *TIFY* genes were identified in the cucumber 9930 genome v2.0 [37], it is essential to identify them based on the cucumber’s pan-genome. Studies have demonstrated that a single reference genome is inadequate to capture the diversity within a species [38,39,40,41]. Furthermore, a pan-genome was constructed by analyzing twelve cucumber genomes [43]. Therefore, we identified and characterized the TIFY family in twelve different cucumber varieties. Fifteen *TIFY* genes were identified in the cucumber 9930 genome v3.0. Consistent with cucumber 9930, fifteen members of *CsTIFYs* were also identified in cucumber Hx14 and Gy14. In cucumber Cuc80, only thirteen members were identified. Additionally, we identified sixteen cucumber *TIFY* genes through a genome-wide analysis of eight other cucumber varieties (Table 1). Among them, two *CsJAZ5* genes were identified and *CsJAZ9* was absent in cucumber W8. Gene duplication is one of the reasons for the expansion of a gene family [46]. The results indicate the possibility of gene replication or deletion occurring during the evolutionary process, which may enhance the adaptability of different cucumber varieties to different cultivation conditions. Upon observation, we identified 71 variants localized within these *CsTIFY* genes, including 56 SNPs and 15 InDels (Table S1). These variants lead to the variations of protein sequence and length, gene structure, and conserved motifs among the same *CsTIFY* genes across different cucumber varieties (Table 2, Figure 2). Therefore, it is suspected that *CsTIFY* genes in different cucumber varieties have undergone distinct evolutionary changes to adapt to diverse environmental conditions.

Both tandem and segmental duplications contributed to the expansion of the gene family [45]. Three segmental duplication and one tandem duplication events within fifteen *CsTIFY* genes were observed (Figure S3 and Table S3), indicating that gene duplication had made some contributions to the *TIFY* gene expansion during the cucumber evolutionary process. No gene replication events were identified for nine *CsTIFY* genes, illustrating that most *CsTIFY* genes might all play an irreplaceable role in cucumber growth and development. Comparative syntenic maps were constructed for cucumber with two monocots (rice and maize) and three dicots (*Arabidopsis*, tomato, and melon) (Figure S4 and Table S4). Only four *CsTIFY* genes (*CsJAZ2*, *CsJAZ3*, *CsJAZ6*, and *CsJAZ8*) displayed orthologous relationships with the ones found in the two monocots and three dicots. In contrast, certain collinear gene pairs (including four *CsTIFY* genes: *CsJAZ7*, *CsPPD1*, *CsZML1*, and *CsTIFY1*) were observed between cucumber and dicots (*Arabidopsis*, tomato, and melon) but not between cucumber and monocots (rice and maize) (Table S4). These results might indicate that orthologous pairs involving *CsTIFY* genes *CsJAZ7*, *CsPPD1*, *CsZML1*, and *CsTIFY1* formed following the divergence of dicotyledonous and monocotyledonous plants, while orthologous pairs with *CsTIFY* genes *CsJAZ2*, *CsJAZ3*, *CsJAZ6*, and *CsJAZ8* arose before the divergence of dicotyledonous and monocotyledonous plants. Additionally, over 85% (13 of 15) of *CsTIFY* genes exhibited orthologous relationships with *TIFY* genes in melon (Figure S4 and Table S4), whereas only four *CsTIFY* genes were found to have orthologous relationships with *TIFY* genes in rice or maize. This suggested that evolutionary rates were similar between the two Cucurbitaceae species, but distinct from those of monocotyledonous species.

### 3.2. Identification of CsTIFYs in Regulating Cucumber Resistance to Gray Mold

A previous study had reported that the expression of JAZ subfamily genes was significantly changed when cucumber plants were infected by gray mold pathogen [37]. In this study, we investigated the expression levels of all *CsTIFYs* after inoculation with the pathogen *B. cinerea* based on published transcriptome data and qRT-PCR analysis (Figure 3 and Figure 4). This finding aligns with previous results that suggest the participation of the *JAZ* subfamily genes in cucumber resistance to gray mold. Additionally, we found that the expression of TIFY, PPD, and ZML subfamily genes was also influenced by the inoculation of gray mold pathogen *B. cinerea* (Figure 3 and Figure 4). Two JAZ subfamily genes (*CsJAZ2* and *CsJAZ6*) and one ZML subfamily gene (*CsZML2*) were selected for functional analysis. As shown in Figure 5, more serious disease symptoms were found in the *CsJAZ2*/*CsJAZ6*/*CsZML2-*overexpressing cotyledons of cucumber seedlings compared to control seedlings. These results suggest that *CsZML2*, *CsJAZ2*, and *CsJAZ6* all affect the cucumber resistance to gray mold, with *CsJAZ6* potentially playing a particularly significant role.

The previous study revealed that a single transition from A to G at position 323 of the *STAYGREEN* (*CsSGR*) gene coding region in Gy14/WI2757 resulted in a higher disease resistance than cucumber 9930 [47]. Several SNPs and InDels of *CsZML2* and *CsJAZ2* genes were observed; meanwhile, we noted variations in the protein sequence and length of them among diverse cucumber varieties (Table 2 and Table S1). It is hypothesized that the functions of *CsZML2* and *CsJAZ2* may vary among different cucumber varieties. Additional research is required to investigate and validate this hypothesis. Regarding the *CsJAZ6* gene, it was found to increase the susceptibility of cucumber seedlings to *B. cinerea* inoculation more than *CsZML2* and *CsJAZ2*. Furthermore, there were no differences in the protein length, gene structure, and conserved domains of the *CsJAZ6* gene among diverse cucumber varieties (Table 2 and Figure 2). These results indicate that *CsJAZ6* is evolutionarily conserved among different cucumber varieties and may play an important role in cucumber plant resistance to disease.

### 3.3. The Involvement of CsJAZ Genes in the JA Pathway Controls Cucumber’s Resistance to Gray Mold

**Figure 7:**
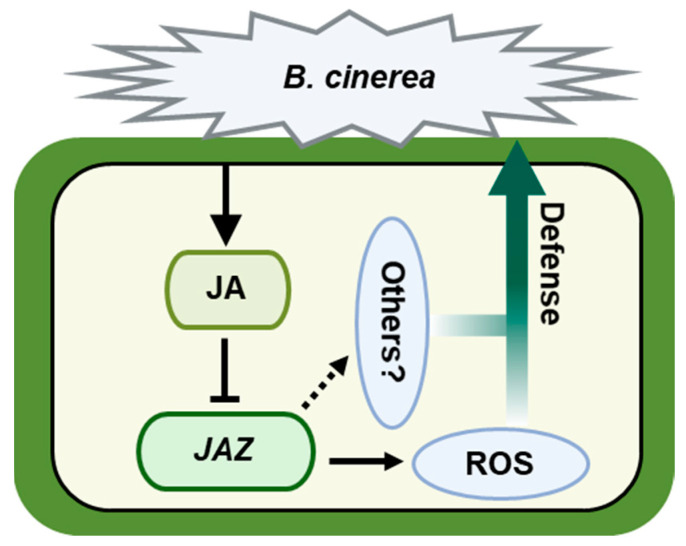
Defense response model of cucumber regulated by *JAZ* genes. After infection with *B. cinerea*, the production of endogenous JA inhibits the expression of *JAZ* genes, thereby affecting the accumulation of ROS to regulate the defense response to *B. cinerea*. Apart from the accumulation of ROS, there might be additional defense signaling pathways that are regulated by *JAZ* genes to affect gray mold resistance.

JAs are phytohormones that play a pivotal role in regulating plant defense mechanisms. The JAZ repressor proteins are central to the signaling cascades activated by JAs. When plants are exposed to stress, the JAZ proteins are degraded by the SCF^COI1^ complex in response to JA-IIe [48]. Considering the important role of JAZ proteins CsJAZ2 and CsJAZ6 in regulating cucumber resistance to gray mold, we analyzed the role of JA in response to gray mold pathogen. It was found that the JA contents in cucumber leaves were significantly increased after inoculation with *B. cinerea* (Figure 6A). Furthermore, pretreatment with exogenous MeJA of cucumber seedlings significantly increased the resistance to *B. cinerea* (Figure 6B,C). Based on these findings, we propose a possible model in which *CsJAZs* induce susceptibility to gray mold disease by repressing the JA pathway to transcriptionally repress the defense genes (Figure 7). In *Rosa chinensis*, JAZ1, which serves as a critical suppressor of JA signaling, is implicated in JA-triggered resistance against pathogens. Specifically, JAZ1 directly interacts with RcMYB84 to impede the expression of *RcMYB123*, thus inhibiting the plant’s defense response against the fungal pathogen *B. cinerea*. However, upon JA treatment, JAZ1 undergoes degradation, leading to the release of *RcMYB84* and *RcMYB123*. Consequently, this activation of defense responses enhances the plant’s resistance to *B. cinerea* [29]. *RcJAZ1* and *CsJAZ2* both were the homolog of *Arabidopsis JAZ1*. These results support the hypothesis that JAZ subfamily genes had a conserved role in plant resistance to gray mold disease by repressing the JA pathway.

Additionally, the enzymatic activities of POD, SOD, and CAT were observed to decrease in the transiently transfected plants that overexpressed *CsJAZ2* or *CsJAZ6* following the inoculation treatment, in comparison to the control plants (Figure 5C). ROS have been proposed as a crucial component in the plant defense response [49]. During plant–pathogen interactions, ROS play a coordinated role in regulating the hypersensitive response [50]. Thus, the accumulation of ROS may play a crucial role in the JA-JAZs resistance mechanism against pathogens in cucumber (Figure 7). Based on previous studies, it has been demonstrated that JAZs participate in multiple signaling pathways to regulate defense responses, such as ethylene signaling pathway [51] and Trp metabolism [52]. Therefore, we speculated that JAZs might also regulate additional defense signaling pathways that impact gray mold resistance. Further studies are necessary to explore and confirm this hypothesis.

## 4. Materials and Methods

### 4.1. Identification and Phylogenetic Tree Construction of TIFY Genes

To identify *CsTIFY* genes in the twelve cucumber genomes (https://www.ncbi.nlm.nih.gov/, accessed on 2 September 2023), including two East Asian lines (XTMC and Cu2), three Eurasian lines (Cuc37, Gy14, and9110gt), one Xishuangbanna line (Cuc80), and five Indian lines (Cuc64, W4, W8, Hx14, and Hx117), the TIFY domain (PF06200) was used for a Hidden Markov Model (HMM) search by HMMER 3.0. The candidate members’ sequences were analyzed using Pfam (http://pfam.xfam.org, accessed on 10 September 2023) and SMART (http://smart.embl-heidelberg.de, accessed on 10 September 2023) to verify the presence of the TIFY domain.

The alignments of aa sequences from members of the TIFY family in *Arabidopsis* and cucumber were conducted using ClustalW in MEGA 7 (7.0.21). These alignments were then used to construct a phylogenetic tree using the Neighbor-Joining (NJ) method. The resulting phylogenetic tree was visualized and enhanced using Evolview (http://www.evolgenius.info/evolview, accessed on 6 October 2023).

### 4.2. Bioinformatics Analysis of CsTIFY Genes

Protein sequences and lengths, gene structure, and conserved protein domains were analyzed. Gene structure was visualized using TBtools based on gene annotation information. The motifs were analyzed by the MEME online program (https://meme-suite.org/meme/tools/meme, accessed on 14 September 2023). The aa sequences were aligned using ClustalW in MEGA 7.0, and this alignment was utilized to construct the phylogenetic tree using the NJ method. The combination images of phylogenetic clustering, conserved protein motifs, and gene structure of *CsTIFY* genes were visualized and optimized using the TBtools.

The cucumber 9930 (v3.0) genomic sequence annotation file was used to visualize the position of TIFY genes on chromosomes through TBtools. To explore the syntenic relationships of the *CsTIFY* genes and other selected species (dicots: *Arabidopsis*, tomato, and melon; monocots: rice and maize), syntenic analysis maps were constructed using TBtools (One Step MCscanX). *Arabidopsis* genomic information was available at The *Arabidopsis* Information Resource (https://www.Arabidopsis.org/, accessed on 20 September 2023), melon genomic information was available at Cucurbit Genomics Database (CuGenDB) (http://cucurbitgenomics.org/, accessed on 20 September 2023), and tomato, rice, and maize genomic information was available at EnsemblPlants (http://plants.ensembl.org/index.html, accessed on 20 September 2023).

### 4.3. Analysis of the Expression Pattern of CsTIFY Genes Based on Published Data

We investigated the expression pattern of *CsTIFY* genes following inoculation with *B. cinerea* using published RNA-seq data [37]. Subsequently, we utilized TBtools software (v2.012) for heatmap generation.

### 4.4. Real-Time PCR Used for Expression Analysis of CsJAZs

After approximately 4 days of in vitro cultivation on a potato dextrose agar (PDA) plate, when the *B. cinerea* grew to a diameter of about 9 cm, the peripheral fungal disks were obtained using an 8 mm puncher. Subsequently, these fungal disks were used to inoculate cucumber cotyledons. Samples were collected at different time points: 0, 6, 12, 24, 48, and 72 h after inoculation. Three biological replicates were taken for each time point, and leaf samples were immediately frozen in liquid nitrogen and stored at −80 °C. TRIzol reagent was used to extract the total RNA. Subsequently, cDNA was synthesized using a reverse transcription kit. The SYBR Green PCR Master Mix was used in the real-time PCR. Sample normalization was performed by the comparative CT method, and the transcriptional level of the gene was normalized to that of the cucumber actin gene. All primers for real-time PCR can be found in Table S5.

### 4.5. Construction of Recombinant Plasmids and Transient Infestation of Cucumber Cotyledons

The coding sequences of three selected *TIFY* genes were inserted into the expression vector pFGC5941 using the *Nco*l and *BamH*I restriction enzyme recognition sites. The recombinant plasmid was transformed into Agrobacterium (strain GV3101) using a freezing–thawing method. Subsequently, *Agrobacterium tumefaciens* carrying the recombinant plasmid was injected into one-week-old cucumber cotyledons. Inoculation with *B. cinerea* was performed 18 h after injection, and the spot area was measured at 24 and 36 h after infection. The area of the lesion was quantified using Digimizer software (5.4.4). All primers can be found in Table S5.

### 4.6. Enzyme Activity Measurement of POD, SOD, and CAT

POD activity was quantified using guaiacol colorimetry, with absorbance readings taken at 470 nm. SOD activity was assessed using NBT, with absorbance measurements performed at 560 nm. CAT activity was calculated based on absorbance readings obtained at 240 nm.

### 4.7. Determination of Plant Endogenous JA Content

At 0 dpi, 1 dpi, and 3 dpi following inoculation with *B. cinerea*, 0.5 g of cucumber cotyledon was flash-frozen in liquid nitrogen and used for detection of endogenous JA content by Enzyme-Linked Immunosorbent Assay (ELISA). The percentage of lesion area was recorded at 1 dpi, 2 dpi, 3 dpi, and 4 dpi, respectively, after applying JA with water as the control.

## 5. Conclusions

In this study, we identified sixteen *CsTIFY* genes based on the pan-genome of twelve cucumber varieties. Bioinformatics analysis results indicate that two *CsTIFYs* showed evolutionary conservation across different cucumber varieties and imply that they may play a crucial role in cucumber growth. On the other hand, the other fourteen *CsTIFY* genes exhibited divergent evolution, possibly because these genes are involved in adapting to various cultivation and environmental conditions. *CsJAZ2*, *CsJAZ6*, and *CsZML2* were found to decrease the cucumber resistance to gray mold and enzymatic activities of POD, SOD and CAT. Additionally, the infection of *B. cinerea* upregulates the content of JA, and treatment with exogenous MeJA increased cucumber resistance to *B. cinerea* infection compared to the treatment with water. In conclusion, our results demonstrate that *CsJAZs* negatively regulate cucumber disease resistance to gray mold via multiple signaling pathways.

## Figures and Tables

**Table 1:** Identification of *TIFY* genes in different cucumber varieties ^a^.

Gene Name	Gene ID ^1^												
	9930-V3	9930-V2	XTMC	Cu2	Cuc37	Cuc64	Cuc80	W4	W8	Hx14	Hx117	Gy14	9110gt
*CsJAZ1*	*1G007260*	*1G042920*	*1G007320*	*1G007500*	*1G007370*	*1G007340*	*1G007330*	*1G007340*	*1G007380*	*1G013550*	*1G010560*	*1G012580*	*1G007620*
*CsJAZ2*	*1G041270*	*1G597690*	*1G046320*	*1G037640*	*1G041300*	*1G052450*	*—*	*1G062110*	*1G039100*	*—*	*1G058680*	*1G049580*	*1G041850*
*CsJAZ3*	*3G030830*	*3G645940*	*3G047350*	*3G036860*	*3G046720*	*3G052270*	*3G042000*	*3G036650*	*3G035010*	*1G044590*	*3G055960*	*3G046570*	*3G037230*
*CsJAZ4*	*4G002460*	*4G009880*	*4G002440*	*4G005390*	*4G002460*	*4G017990*	*4G002380*	*4G002450*	*4G002460*	*1G056790*	*4G002460*	*4G003410*	*4G003440*
*CsJAZ5*	*5G037080*	*5G628650*	*5G058190*	*5G053120*	*5G055380*	*5G042390*	*5G061610*	*5G039340*	*5G044750* *UNG162140*	*2G021820*	*5G066040*	*5G060960*	*5G045250*
*CsJAZ6*	*6G007840*	*6G091930*	*6G009050*	*6G008870*	*6G007910*	*6G008010*	*6G012070*	*6G007970*	*6G009990*	*2G038620*	*6G008030*	*6G012110*	*6G007990*
*CsJAZ7*	*6G051810*	*6G523460*	*6G066600*	*6G047700*	*6G048270*	*6G046340*	*—*	*6G045210*	*6G045460*	*2G038630*	*6G057100*	*6G058030*	*6G049490*
*CsJAZ8*	*7G034270*	*7G448810*	*7G042890*	*7G033210*	*7G046090*	*7G037890*	*7G045520*	*7G031980*	*7G045180*	*2G041060*	*7G049570*	*7G041480*	*7G034610*
*CsJAZ9*	*—*	*—*	*1G035110*	*1G028480*	*1G033150*	*1G042040*	*—*	*1G029160*	*—*	*3G052170*	*1G042630*	*1G038520*	*1G030630*
*CsZML1*	*2G030170*	*2G370420*	*2G031610*	*2G030370*	*2G100170*	*2G070540*	*2G103280*	*2G035530*	*2G042710*	*3G075320*	*2G041600*	*2G038450*	*2G031870*
*CsZML2*	*2G030180*	*2G370430*	*2G031620*	*2G030380*	*2G100180*	*2G070550*	*2G103290*	*2G035540*	*2G042720*	*5G059690*	*2G041610*	*2G038460*	*2G031880*
*CsZML3*	*7G006810*	*7G064580*	*7G010060*	*7G006730*	*7G005610*	*7G002370*	*7G005770*	*7G005620*	*7G010880*	*6G011910*	*7G010800*	*7G006710*	*7G007870*
*CsZML4*	*7G033740*	*7G447800*	*7G042380*	*7G032680*	*7G045570*	*7G037360*	*7G044990*	*7G031470*	*7G044650*	*6G064770*	*7G049040*	*7G040950*	*7G034070*
*CsTIFY1*	*2G031660*	*2G379290*	*2G034120*	*2G032830*	*2G101610*	*2G071990*	*2G104740*	*2G037020*	*2G044220*	*7G008850*	*2G044080*	*2G039930*	*2G033380*
*CsTIFY2*	*3G046630*	*3G878900*	*3G069390*	*3G057580*	*3G062760*	*3G068300*	*3G058900*	*3G053660*	*3G052280*	*7G043050*	*3G073290*	*—*	*3G054610*
*CsPPD1*	*2G013410*	*2G222060*	*2G013060*	*2G014060*	*2G011860*	*2G011850*	*2G015800*	*2G014790*	*2G020910*	*7G043570*	*2G017780*	*2G019980*	*2G015010*

“^a^” *TIFY* gene (1G435720), only identified in 9930 cucumber genome v2.0, was not presented in table. “^1^” The gene IDs in the table did not include abbreviations that could represent different cucumber varieties; “—” indicates the *TIFY* gene was not identified in this cucumber cultivar.

**Table 2:** The predicted lengths of TIFY proteins in different cucumber cultivars.

Protein Number	9930-V3	XTMC	Cu2	Cuc80	Cuc64	W4	W8	Hx14	Hx117	Cuc37	Gy14	9110gt
CsJAZ1	339	340	340	356	356	340	340	340	340	340	340	340
CsJAZ2	231	231	169	—	231	231	231	231	231	231	231	231
CsJAZ3	209	209	209	209	209	209	209	209	209	209	209	209
CsJAZ4	200	200	200	200	190	200 *	200	—	190	200	190	200
CsJAZ5	381	381	381	381	381	381	381	381	381	381	381	381
CsJAZ6	184	184	184	184	184	184	184	184	184	184	184	184
CsJAZ7	132	132	132	—	130	130	130	132	132	132	132	132
CsJAZ8	295	295	295	295	295	295	295	295	295	295	295	295
CsJAZ9	—	150	152	—	150	152	—	154	154	152	152	107
CsZML1	352	352	352	352 *	352	352	352	352	352	352	352	352
CsZML2	279	293	293	293	293	293	293	293	293	293	293 *	293
CsZML3	313	321 *	321	321	321	321	316	313	313	321	321	321
CsZML4	303	303	303	332	303	303	303	303	303	303	303	303
CsTIFY1	274	274 *	274 *	274 *	274 *	274 *	274 *	274 *	274 *	274 *	274 *	274 *
CsTIFY2	376	400	400	400	400	400	400	400	376	400	—	400
CsPPD1	336	336	336	336	336	336	336	336	207	336	305	336

The proteins that have differences in length compared to *Cucumis sativus* 9930 are marked in red; “—” indicates the TIFY gene was not identified in this cucumber cultivar; “*”, gnome with assembly error, the corrected protein length is shown in the table.

## Data Availability

Data is contained within the article and Supplementary Materials.

## Supplementary Materials

The following supporting information can be downloaded at: https://www.mdpi.com/article/10.3390/ijms25010185/s1.
