# Supplementary material for: Pan-Genome Analysis of TIFY Gene Family and Functional Analysis of CsTIFY Genes in Cucumber
Source: Int J Mol Sci. 2023 Dec 22;25(1):185. doi: 10.3390/ijms25010185 (PMC10778933; doi:10.3390/ijms25010185)
Supplement: Supplementary file 1 [file ijms-25-00185-s001.zip › Suplemental figures.pdf]

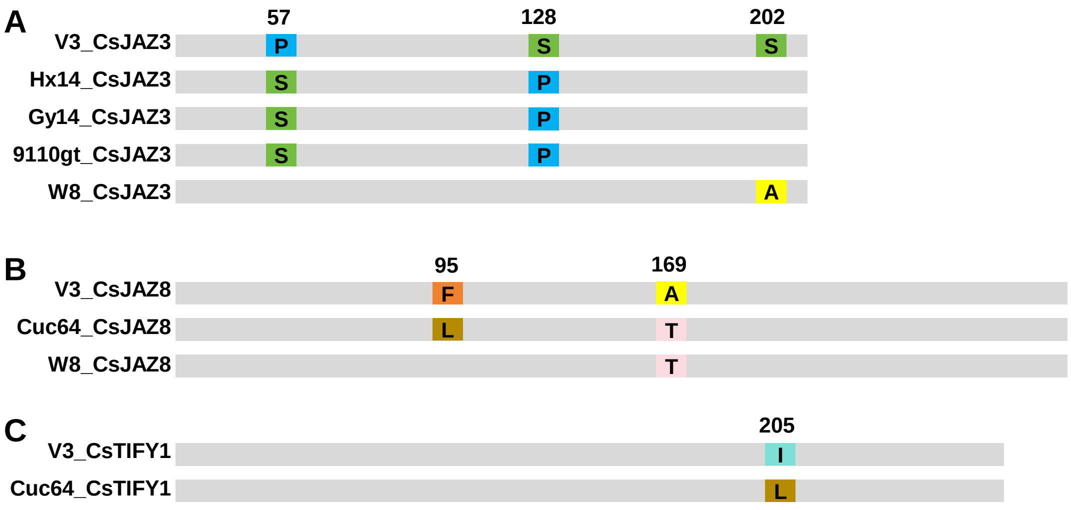


Figure S1. Sequence diversity within the TIFY proteins among different varieties. The comparation of protein sequences of CsJAZ3 (A), CsJAZ8 (B), and CsTIFY1(C) between cucumber 9930 and other cucumber varieties.

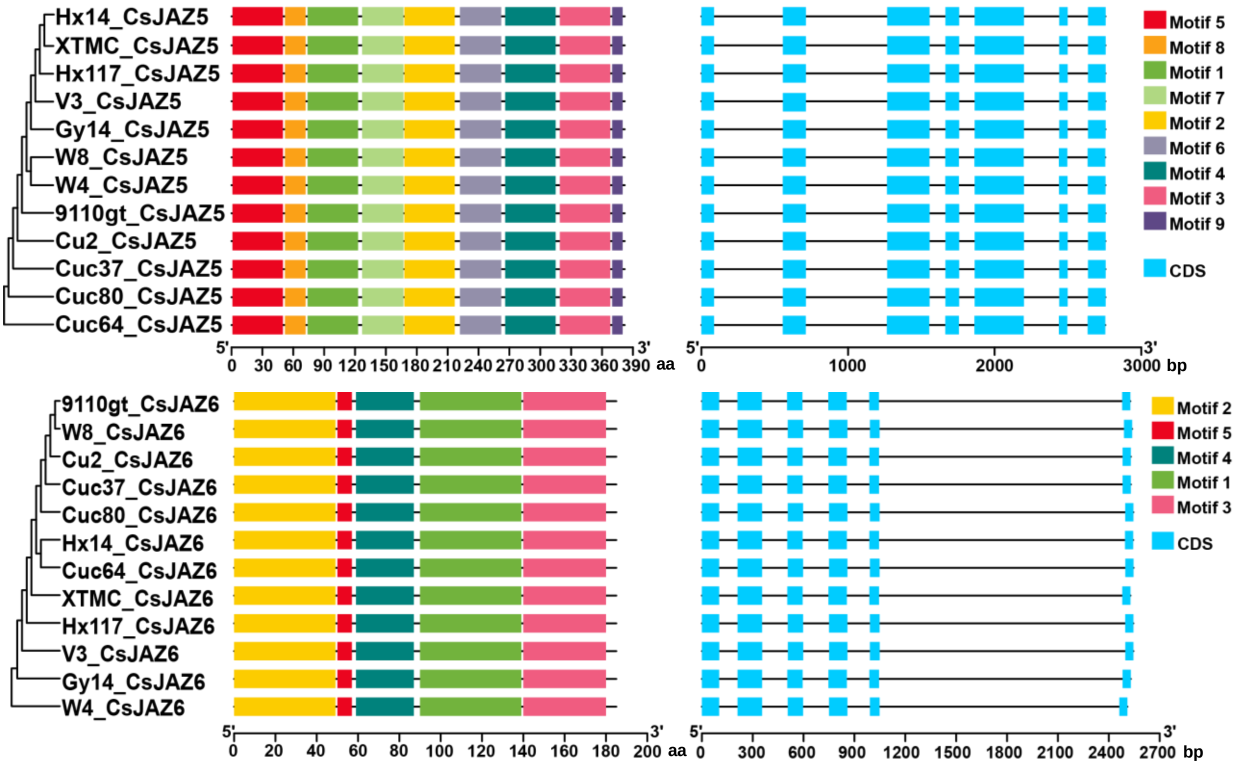


Figure S2. The phylogenetic tree, conserved protein motifs, and gene structure of *CsTIFY* genes, which code for proteins of the same sequence and length. Right panel: gene structure, blue squares indicate CDS regions and black lines indicate introns. Middle panel: conserved protein motifs. The colorful boxes delineate different motifs. Left panel: the phylogenetic tree. The clustering is performed according to the results of phylogenetic analysis.

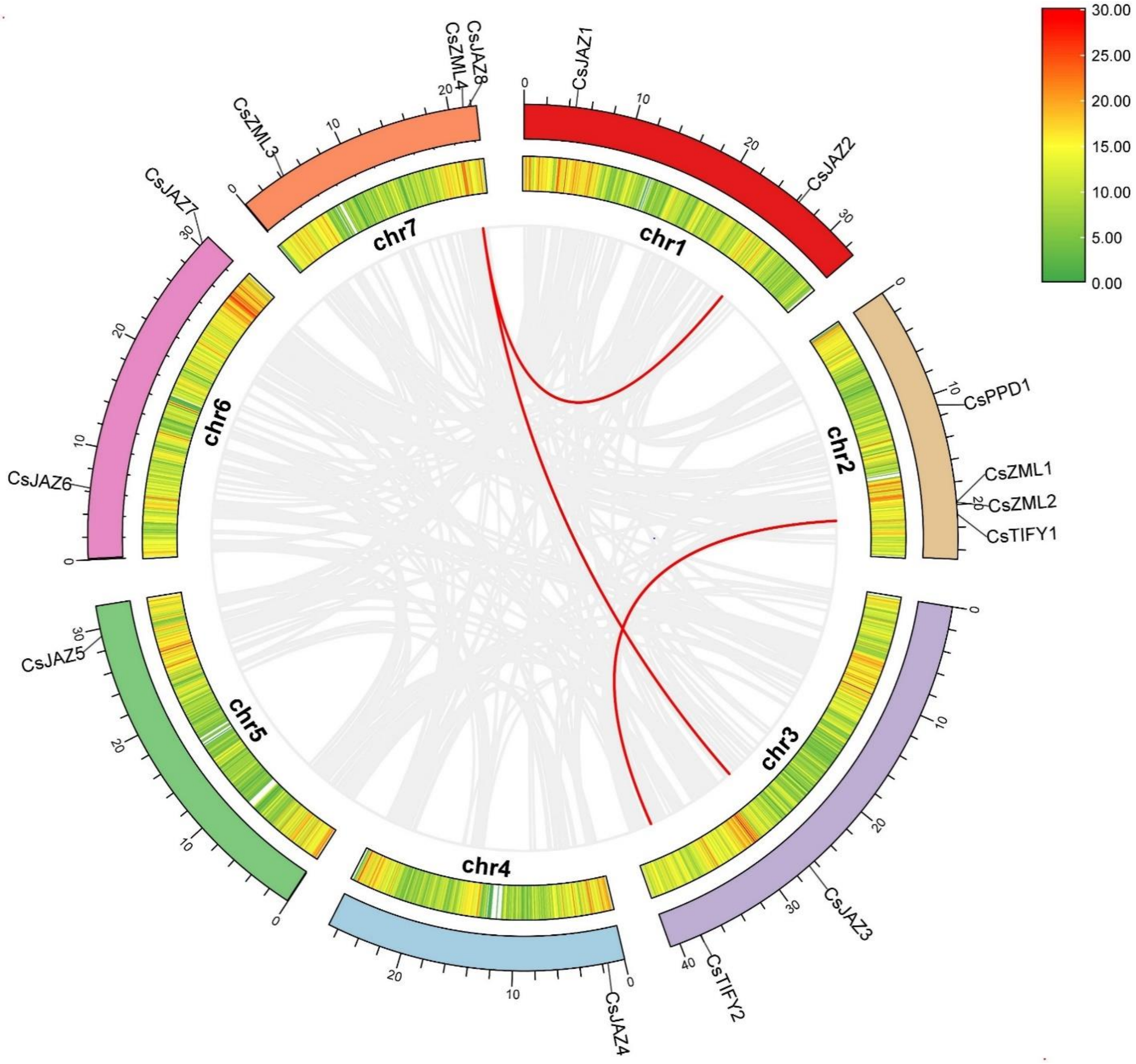


Figure S3. Schematic representations for the interchromosomal relationships of *CsTIFYs*. Gray lines show synteny blocks in the cucumber genome,duplicated *TIFY* gene pairs are connected with red lines.

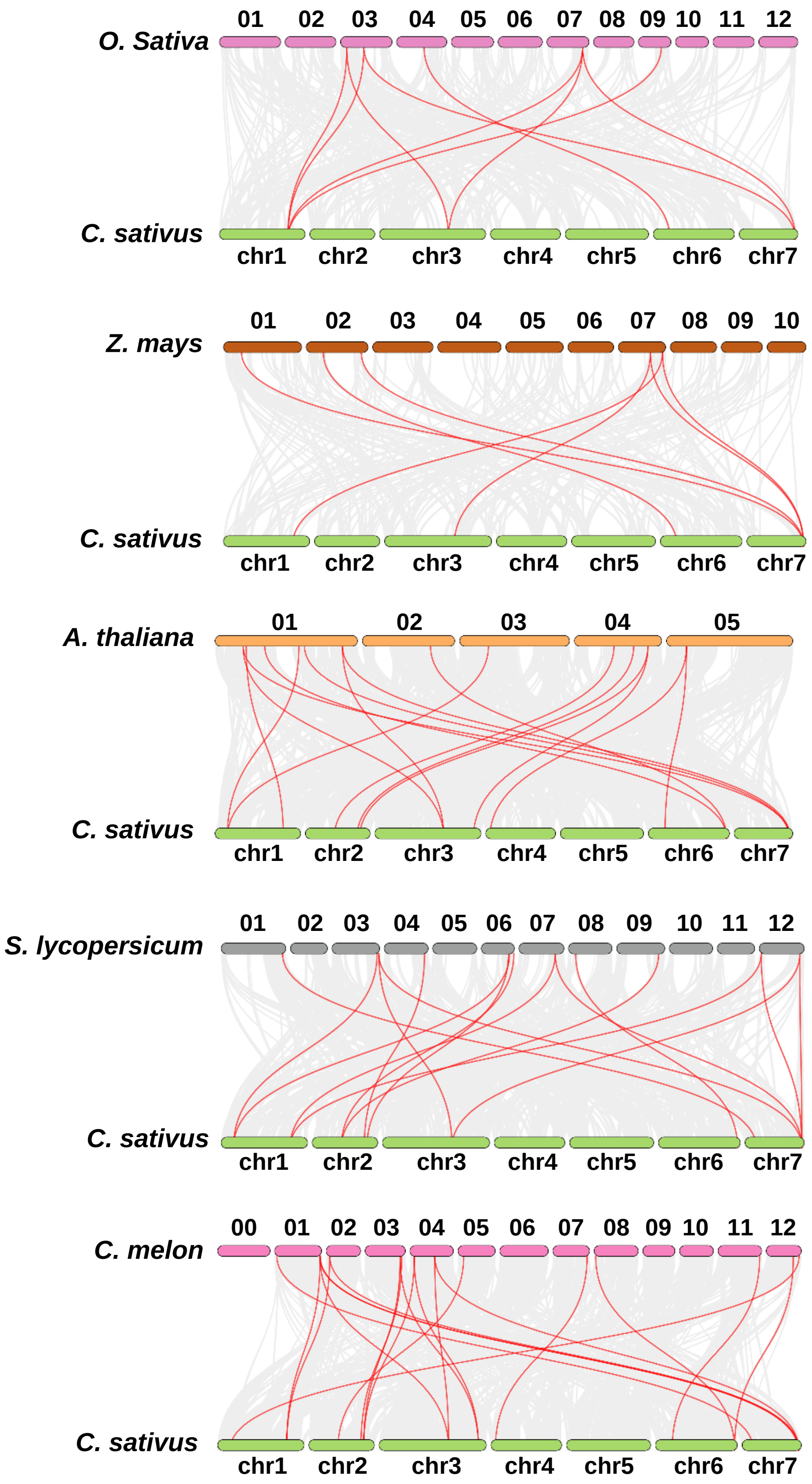


Figure S4. The synteny analysis of *TIFYs* between cucumber and other plant species. Collinear gene pairs with *TIFY* genes are marked by red lines. '*C. sativus*', '*O. sativa*', '*Z. mays*', '*A. thaliana*', '*S. lycopersicum*' and '*C. melon*' indicate *Cucumis sativus, Oryza sativa, Zea mays, Arabidopsis thaliana*, *Solanum lycopersicum*, and *Cucumis melon*, respectively
