# Supplementary material for: Pan-Genome Analysis of TIFY Gene Family and Functional Analysis of CsTIFY Genes in Cucumber
Source: Int J Mol Sci. 2023 Dec 22;25(1):185. doi: 10.3390/ijms25010185 (PMC10778933; doi:10.3390/ijms25010185)
Supplement: Supplementary file 1 [file ijms-25-00185-s001.zip › Table S5.pdf]

Table S5. Primer information used in this study

Primers for qRT-PCR (5’-3’)	
Actin-F	GATTCTGGTGATGGTGTGA
Actin-R	CGATGAGAGATGGCTGGAATA
CsJAZ2-F	AAGAGGCAGGATTTGGTA
CsJAZ2-R	AGCGACGGTAGGGATGTT
CsJAZ3-F	CGGACTCTATTCCACCAG
CsJAZ3-R	ACGGCGCAATTCCCTTAC
CsJAZ6-F	AATCTGTCATTGGCTCTG
CsJAZ6-R	CAAGTGTTGCTGGTGGTC
CsJAZ8-F	AGCAGCAACTGAAACCAC
CsJAZ8-R	TGAGATCCAGATGCCTTG
CsZML2-F	CATTCCGTGGTCAGGTTT
CsZML2-R	CTGTCCTTTGTTGCGTTG
CsZML3-F	ATCCTCAGATGCGGTTCG
CsZML3-R	CAGCGTAAGTTGGTCAGC
**Primers for gene cloning (5’-3’)**	
CsZML2-F	ATGTACGCACGCGCTC
CsZML2-R	AAGCAGCCGTATCA
CsJAZ2-F	ATGTCGACTTCTTCCGGA
CsJAZ2-R	TTTTTGAGACTGCACCGC
CsJAZ6-F	ATGGCTAGGGCAACTG
CsJAZ6-R	AGTTAGGTGGGGGCA
